# Human behavior and performance in deep space exploration: next challenges and research gaps

**DOI:** 10.1038/s41526-023-00270-7

**Published:** 2023-03-30

**Authors:** Francesco Pagnini, Dietrich Manzey, Elisabeth Rosnet, Denise Ferravante, Olivier White, Nathan Smith

**Affiliations:** 1 Department of Psychology, Università Cattolica del Sacro Cuore, Milan, Italy; 2 Department of Psychology and Ergonomics, Technical University Berlin, Berlin, Germany; 3 Faculty of Sport Sciences, Performance, Health, Metrology, Society (PSMS) Laboratory, University of Reims Champagne-Ardenne, Reims, 51096 France; 4 Antarctic Technical Unit, ENEA, Roma, Italy; 5 Faculty of Medicine and Psychology, University of Rome la Sapienza, Roma, Italy; 6 Université de Bourgogne-Franche Comté, Dijon, France; 7 Protective Security and Resilience Centre, Coventry University, Coventry, UK

**Keywords:** Human behaviour, Human behaviour

## Abstract

As space exploration missions move from low orbit to distant destinations, including the Moon and Mars, new psychological, behavioral, and team challenges will arise. This manuscript represents an up-to-date white paper developed by European experts invited by the European Space Agency (ESA), mapping unfilled research gaps related to the psychology of space exploration, considering the incoming human missions, and accounting for the available scientific knowledge. ESA created the expert team and facilitated its work, but the team was completely independent in terms of contents. The white paper considers basic issues of adaptation, pre-, during-, and post-mission experiences, and possible countermeasures to be developed and tested. The resulting integrative map provides a guide for researchers that are interested in conducting research in the support of future space exploration endeavors.

## Introduction

The next phase of human space exploration includes the establishment and habitation of a lunar gateway, a permanent base on the Moon’s surface, and exploratory crewed missions to Mars. As human activity in space moves from Low Earth Orbit (LEO) operations, such as those that take place on the ISS, to deep space exploration, the crews will face a different set of psychological challenges^1^. These include extended mission durations, increased distance from Earth, prolonged isolation and confinement, reduced size of crew quarters, lack of privacy, communication latency, need for increased autonomy in decision-making processes, and lack of short-term rescue possibilities, amongst other known and as yet unknown demands^2,3^.

There is good evidence to suggest that astronauts’ behavior, health, and performance can be impacted by the demands encountered in future space missions^4^. For the purposes of this work, issues of behavior, health, and performance are separated and considered according to discrete cognitive, affective, behavioral, social, and mental health components. While these function areas are related, distinguishing between them provides the opportunity for conducting well-specified and targeted psychological studies. Cognitive dimensions include issues of perception, vigilance, judgment, memory, and reaction time, amongst other executive functions^5^. When discussing cognition, there are clear synergies with the neurosciences. Affective experiences refer to emotions, feelings, and moods, which can be shaped by a person’s physiology and subjective experience. Behaviors include observable individual and interpersonal actions and the execution of skilled performances. For instance, before they are executed, motor actions must be planned in the brain and rely on complex neuronal networks. Team-level social functions include relevant team process dynamics, such as experiences of cohesion and conflict. Mental health is a relevant component, which is related to the psychosocial, affective, cognitive, emotional, and physical challenges that astronauts face during missions. Mental health includes the importance of managing psychophysical stress and promoting well-being, both on individual and team levels.

This white paper is the result of a consensus among experts invited by the European Space Agency to update the roadmap for scientific research for the next decade^6^. The psychology working group (corresponding to the authors of this paper) was composed of experts in psychological science with a large research experience in the space context, currently engaged in research with ESA, in the space environment, analogue environments, and ground-based research. Research gaps were identified by the experts, referring to their direct research experience and to their knowledge of the scientific literature, and discussed to reach a consensus. The work specifically focused on the behavioral and performance aspects. Some of the relevant aspects that could impact the astronauts’ life, including the effects of space radiations, were not addressed by this group, as they have been included in other working groups’ report.

In this report, we broadly refer to long-duration (i.e., >30 days) space exploration (LDSE) missions, with a particular focus on deep space voyages, outside the earth’s atmosphere, which are distinct from current long missions in LEO. Even with this differentiation, it is worth keeping in mind that the psychological challenges of relatively near-Earth explorations, including incoming Moon missions, may be qualitatively different from those that will be experienced during long-distance deep space voyages, such as human missions to Mars—which also include long travels and an extreme routine. In the following, many of the open questions related to psychological function in space are framed in the context of LDSE missions. However, where we refer to space missions broadly, questions are pertinent to LEO, lunar and deep space missions. With respect to an understanding of human performance and behavior issues of spaceflight, the European Space Agency and its partners can build on several years of studies and experiences on the ISS and sub-orbital flights, as well as during simulations studies and in analogue environments. Deep space exploration, though, has some different characteristics that will require ad-hoc preparation and new studies to answer currently open questions. For this reason, further studies will be needed not only to be conducted during long-duration space missions, but also in other settings, including analogue environments and other isolated and confined settings. These environments share some similarities with the space context, including isolation and confinement. Some “analogue missions” are conducted in specifically designed facilities, such as HERA (Human Exploration Research Analog), the underwater research station NEEMO (NASA Extreme Environment Mission Operations), and the MARS500 isolation chamber. Other facilities include Antarctic stations, such as the Concordia base, an Italian/French research facility considered one of the most remote outposts on Earth. Most of these environments allow to study behavioral, physical, and team dynamics, and to test countermeasures that can be implemented in space missions. Other analogue environments allow to recreate specific challenges of space research, including radiations (e.g., the NASA Space Radiation Lab) and bedrest implications (e.g., the*: envihab* facility, in Cologne, Germany).

Risks to behavior, health, and performance during deep space exploration could be mitigated and astronaut function optimized with the application of effective countermeasures. However, research is required to identify and further develop or refine the strategies and approaches that might be used to enable astronauts to maintain elevated well-being and high-performance standards on LDSE missions. In the following section, we distinguish between questions related to basic issues of adaptation and countermeasures. The former largely deals with the understanding of psychological aspects of deep space exploration and the impact of unique deep space mission demands upon behavior, health, and performance. This includes the role of individual differences in adaptation, and broader mechanisms underlying individual and team phenomena that are relevant to human spaceflight^7–9^. Important information in this area could be inferred from different types of observational and correlational studies. Countermeasures deal with the specific actions and interventions that space agencies can enact to mitigate the risks of future missions. This might include a refined selection process or the application of inflight psychological training and support. While most questions in the white paper focus on pre-mission and inflight activities, it is also important to consider psychological experiences in the post-mission phase, to ensure that astronauts’ well-being is robust after the end of what are likely to be physically and psychologically demanding voyages. Astronauts constitute a very limited community on Earth. When addressing these fundamental questions, it is therefore critical to consider whether and how the findings can be transferred to the general public as many activities parallel, to some extent, what space travelers will undergo.

### Key knowledge gaps

Most knowledge in space psychology has focused on short-duration mission, relatively close to Earth, and with synchronous contacts with mission control. New incoming space missions pose different challenges, in terms of psychological adaptation and the definition of countermeasures to mitigate risks.

### Basic issues of adaptation

To inform the identification of effective preparatory and preventative countermeasures for future space missions, there are several questions related to basic issues of psychological adaptation that need to be resolved. These questions relate to both in-mission dynamics influenced by the interaction between individual and team factors and contextual demands, and what happens in the post-mission phase. Open questions identified by the expert scientific group, largely reflect unknowns associated with missions beyond Low Earth Orbit (LEO).

#### In-mission

##### Critical markers

For the ISS, there already exists a standard human behavior and performance competency framework for crewmembers^10^. There have also been efforts to standardize the psychological/psychosocial and behavioral data collected during space agency sponsored research activities^11–13^. This research has been used in ground-based studies in analogue environments, such as NASA-HERA, VaPER, AGBRESA, SIRIUS 19 & 20, as well as Antarctica. Standard measures have been used on the International Space Station in multiple expeditions^14^. These standard measures have included cognition batteries, personality surveys, and neuroscience assessments of sensorimotor measures, together with biomarkers, immune markers, and actigraphy. All these data will help create useful benchmarks for LDSE missions. Nevertheless, despite the important research and the progress made so far, there is a need to continue this research in analog environment and long-term spaceflight. In addition, the scope of this research should be enlarged with respect to including also physiological measures as possible indicators of mental health and performance state, as well as measures of team cohesion and climate which can capture team dynamics in the course of LDSE missions. Questions that must be addressed include:

- What variables capture relevant information on cognitive, affective, behavioral function, and team functions? And what outcomes are informative about mental health issues?
- What physiological biomarkers provide a valid insight to the psychological experience of astronauts?

#### Stressors

To date, there have been many studies on spaceflight stressors that have utilized both space platforms and ground-based analogs. However, for LDSE missions the type and magnitude of stressors encountered will be different from those experienced in the past. Research is needed to evaluate the impact of such stressors on individual and team function. Certain stressors could be evaluated in analog environments (e.g., isolation, confinement, chronic threat, sleep deprivation, sensory deprivation), while the effects of others will need to be examined in space (microgravity, hypogravity). Research conducted during spaceflight, in analog environments and simulation studies, thus far, has already provided important insights into the effects of microgravity, prolonged confinement, and isolation, as well as sleep deprivation on human performance, well-being, and behavior^15^. However, the impact of stressors such as prolonged hypogravity, transitions between different levels of hypogravity, prolonged radiation exposure, chronic threat due to lacking rescue possibilities, extreme levels of crew autonomy, and Earth-out-of-view unique to LDSE missions, have not been addressed yet, or only to a very limited degree (e.g., effects of autonomy during simulation studies)^16,17^. Their impact on cognition, affective experiences, behavior and performance, team dynamics, mental health, and performance should be carefully assessed. Some of these stressors can be studied in analog environments (e.g., autonomy), others require spaceflight experiments during upcoming missions to the Moon (e.g., hypogravity) and still others might be only investigated during actual LDSE missions (e.g., Earth-out-of-view effects). Moreover, the relationship among stress biomarker dynamics (e.g., heart rate variability, cortisol), related biological processes, and individual and team function within these settings will have to be clearly unfolded.

##### Individual and team characteristics

While there have been many studies on individual differences in relation to human spaceflight, data that exist remains relatively limited in a predictive sense^1^. Additional research is needed to examine how baseline individual and team characteristics are likely to impact upon in-mission individual and team function. Moreover, day-to-day team performance indicators need further exploration in the context of extreme environments.

- How do demographic criteria (e.g., age, gender identity, and ethnicity) influence adaptability and individual function during space missions?
- What is the unique impact of sexuality on the psychological function of crewmembers in space?
- How do individual psychological differences (e.g., personality, motivation, and values) influence adaptability and individual and team function during space missions?
- What individual difference factors should be used to inform effective team composition decisions for LDSE missions?
- How do team structure and composition influence crew adaptability and function during space missions?
- How do social dynamics, values, norms, and culture influence crew adaptability and function during space missions?
- How can we assess team performance and dynamics on a “small scale”, and how does that relate to the overall mission success?

##### Coping and regulatory strategies

The high levels of autonomy that will be encountered on LDSE missions mean that crew will have increased responsibility for their own self-care/self-management^18^. New research is needed to examine the coping and health and performance self-regulation strategies that individuals and teams use to maintain their function. Although there have been initial studies on coping in analog settings^19^, there is limited empirical data on what astronauts do to manage themselves and their teams during space missions (LDSE or otherwise). Open questions are:

- What resources and equipment (e.g., food variety, entertainment systems) contribute to effective coping and self-regulation during LDSE missions?
- What coping and regulatory strategies are effective for optimizing cognition, affective experiences, behavior and performance, individual and team function, and, more in general, to promote mental health during LDSE missions?

##### Interactive effects

Integrative studies that examine the interactive effects of psychosocial factors alongside physiological responses and other features of the environment, such as habitat design and resource availability, are required to provide a deeper insight to the human experience in space. This is especially important for LDSE where new systems, equipment, and habitats will be used. Among the unanswered questions, we find:

- How do spaceflight stressors, demographic criteria, individual differences, coping, and regulatory strategies interact to impact individual and team function during LDSE missions?
- What factors predict the extent to which skill fade occurs during LDSE missions?
- How do physical features of the environment (e.g., habitat, architecture, internal conditions, and plants) impact upon individual and team adaptation?
- How do food perception (e.g., taste and olfaction), texture, and variety impact upon astronauts’ affective, experiences?
- How do astronauts interact with reporting systems designed to capture safety-critical information (e.g., medication use)?
- What individual and team factors impact upon compliance with reporting systems designed to capture safety-critical information (e.g., medication use)?

#### Post-mission

The post-mission phase has addressed by research one both overwinter missions in Antarctica (e.g., ref. ^20^), and NASA post-flight standard measures^14^. However, it still requires a structured and deepened exploration, which has been sometimes overlooked. Anecdotal reports from the astronauts of the lunar missions in the 1960ies and 70ies suggest, that the mental processing of such extreme experiences represents a challenge also after the actual mission. With LDSE missions, the importance of questions related to reintegration, recovery, and mental processing of the mission experiences will significantly raise. Specifically, crucial open questions which need to become addressed more systematically relate to what positive or negative after-effects might occur after prolonged spaceflights, and what regulatory strategies might be effective to support reintegration, recovery, and rehabilitation upon return from LDSE missions. For example, there is limited empirical information on how individuals cope during their return from space and what strategies they use to maintain their health and well-being during reintegration and recovery. Research is needed to identify the strategies that individuals use and what impact that has upon their cognition, affect, and behavior in the post-mission phase. Open questions include:

- What individual coping and regulatory strategies are effective for optimizing cognition, affective experiences, behavior, and performance, and, more in general, mental health, during the return, transition, and recovery following LDSE missions?
- How do social networks contribute to effective astronaut coping and self-regulation during their return?
- How do individuals prepare themselves and their families to redeploy on new missions?

### Countermeasures

Addressing open questions related to basic issues of adaptation should provide the knowledge to develop effective countermeasures for the envisaged future space missions. Psychological countermeasures might target selection and training, in-mission, and post-mission phases. The emphasis in this white paper is on identification and testing and evaluating the impact of applied measures.

#### Selection and training

Current selection and training protocols have been designed for LEO missions. Research is needed to identify how individual and team psychological selection should be adapted for LDSE missions. Specifying and developing the training needed to ensure optimal crew function on LDSE is also needed. While existing processes might continue to have utility, this should be confirmed with empirical evidence. Questions that still have to be addressed include:

- What individual difference factors inform on psychological suitability for LDSE missions?
- How should psychological suitability be assessed during the assessment and selection of astronauts for LDSE missions?
- What methods are available to inform the selection of psychologically compatible or incompatible teams?
- Do these methods raise any ethical concerns?
- How should current selection processes be adapted and validated to inform the effective psychological selection of crewmembers for LDSE missions?
- What unique training protocols need to be developed and how should they be delivered (e.g., what strategies, tools, and techniques) to prepare individuals and teams to respond effectively to the demands of LDSE missions?
- How should individuals and teams be trained to respond effectively to critical or off-nominal incidents when operating autonomously in space? What protocols and policies need to be developed?
- How should approaches and methods for optimizing affective experiences and cognition (e.g., mind-body strategies, emotion regulation, and flexible coping) during space missions be trained?
- How should approaches and methods for optimizing team function (e.g., communication, cooperation, collaboration, and conflict resolution) during space missions be trained?
- How should astronauts be trained to deal with extreme and unexpected events (e.g., deaths and psychiatric issues) that might occur during LDSE missions?

#### Crew support

Support during and after LDSE missions will rely on accurate monitoring, diagnosis, and deployment of effective countermeasures. Although research in these areas is currently being undertaken, there remain a number of open questions about how to best support individuals and teams in space. Studies conducted in microgravity and on ground-based analogs can be used to identify and evaluate the efficacy of approaches to support individuals and crew during and after return from LDSE missions.

- What methods, measures, and metrics should be used to monitor individual and team function, sleep, and fatigue during space missions?
- How should work/life balance be managed during different phases of a LDSE mission?
- How can astronauts be supported and what resources do they need to allow them to rest and relax away from work tasks?
- How should sleep and fatigue management skills for LDSE missions be trained and maintained?
- What non-pharmaceutical approaches are effective for sleep and fatigue management during LDSE missions?
- How should methods used to minimize skill fade and degradations in task performance during LDSE missions be administered?
- How should astronauts be supported to maintain their motivation to engage in healthy behaviors (e.g., exercise) across the duration of a LDSE mission?
- What and how should support be provided following the occurrence of extreme and unexpected events (e.g., deaths and psychiatric issues)?
- How should approaches and methods for optimizing mental health, affective experiences, cognition, behavior, and performance (e.g., mind-body strategies, emotion regulation, and flexible coping) during space missions be maintained?
- How should approaches and methods for optimizing team function (e.g., communication, cooperation, collaboration, and conflict resolution) during space missions be maintained?
- How should autonomous and digital systems be used to effectively support individual and team functions during LDSE missions?
- How do human factors impact upon autonomous and digital system interaction?
- What features must be included in autonomous and digital systems for effective use in space?
- How do trust and privacy impact the likelihood of astronauts engaging with autonomous and digitally delivered countermeasures?
- What communication types/methods are effective as a mechanism for support during autonomous missions?
- How should communications be adapted to effectively support team function during autonomous LDSE missions?
- What family support mechanisms need to be established to minimize potential issues due to separation and lack of family contact during LDSE missions and what would be the optimal communication frequency and duration?
- How should families and social groups be effectively-prepared to support those returning from space?

##### System design

Psychosocial function of astronauts can be impacted by the system that the individual and team are operating in. The constraints of LDSE missions mean that new systems, architectures, and habitats will need to be developed. There are open questions about how to engineer and design the systems, architectures, and habitats to facilitate optimal function in space:

- How should autonomous and digital systems be designed for use during LDSE missions? In particular, what would be the benefits of using virtual reality-based approaches?
- How should communications be designed to effectively support individual functions during autonomous LDSE missions?
- What architectural and habitat design features should be implemented to enhance individual and interpersonal function during LDSE missions?
- What features should be considered and designed into safety-critical reporting systems (e.g., medication reporting systems)?
- How might an astronaut’s connection to nature be established through architecture and habitat design?

### Priorities for the space program

Several of the identified knowledge gaps have direct relevance for micro- and hypogravity research. In particular, this holds true for a better understanding of the effects of hypogravity on human cognition and performance, which are already relatively well understood for some basic cognitive functions, but which lack knowledge with respect to higher executive functions or issues related to skill maintenance across different levels of (hypo-)gravity. The clear majority of the key knowledge gaps previously identified, however, relate to basic issues of individual or crew adaptation to long-term confinement and isolation and to effective countermeasures for maintaining well-being and performance of crews under such conditions. To close these knowledge gaps is of most relevance for future exploration missions to the Moon and Mars which will involve more extreme conditions of isolation and confinement than has been known from other environments, thus far. Even though the conditions of travel to the Moon will be more extreme than what we know from near-Earth orbital spaceflight and overwintering in Antarctica, they do not seem to be different in a qualitative sense (i.e., the demands are amplified rather than being especially unique). Thus, it might be expected that at least some of the current knowledge about the psychological effects of isolation and confinement as well as hypogravity might be generalized to missions in lunar orbits or even stays on the lunar surface. In contrast, future deep space missions to Mars will represent a qualitatively much more extreme change (e.g., with respect to autonomy, restricted means of crew-ground communication, lack of evacuation possibilities) compared to what has been known about effects of isolation and confinement from other fields already, and, thus, will provide completely new psychological challenges which currently are not well understood. In a sense, future missions to Mars will resemble past ambitious naval explorations, such as those conducted by Vespucci and Colombo, in which humans pushed their limits beyond a line that had never been crossed before^21^. However, on the other hand, we are arguably more prepared than a crew on a ship that did not know what they were about to find, as, we can prepare such missions using probes, satellites, and many other remote observation techniques. Among the preparation activity, psychological research addressing the knowledge identified gaps will be a fundamental step in any space program focusing on exploratory human missions to Mars and beyond. While microgravity and hypogravity pose serious challenges to the central nervous system^22^, most of the knowledge gaps about behavioral and psychological aspects are not related. Therefore, the research needed does not necessarily involve research during actual space missions. Naturalistically extreme environments on Earth (e.g., Antarctica) and, even better with respect to experimental control, simulation studies on the ground will, in many cases, provide appropriate environments for such research.

The research gaps highlighted in this report are in line with the ones identified by NASA^23^. The need to identify and validate countermeasures to promote health and performance, to define improved monitoring and assessment strategies, and to investigate and optimize team dynamics, for example, are shared concerns between this report and the NASA’s Evidence Book for Risk of Adverse Cognitive or Behavioral Conditions and Psychiatric Disorders^23^. Similar conclusions have been described in a recent NASA report^24^ about team research, highlighting the lack of data availability from the space context, and the need for further research on the topic, including studies in analogue environments and subject matter expert interviews.

### Benefits for Earth and industrial relevance

Space travels magnify the challenges posed to a team of astronauts, such as confinement, and lack of external communication. However, there are also many situations that regular workers can face on the Earth and that include—although to a lesser extent—, some features astronauts can meet. For example, teams sometimes work in remote places, where communication is constrained. Therefore, more classical Earth-based activities can benefit from the transfer of this fundamental research.

Research conducted to fill knowledge gaps identified in the psychological phenomenon linked to space exploration may be applied to optimize the behavior, health, and performance of crewmembers in these extreme conditions. Once the processes that might contribute to the possible impairment have been identified, it could be envisaged to elaborate specific countermeasures that could help crewmembers to maintain and enhance their health and performance. For example, innovative and new technologies like virtual reality may be stimulated by this kind of challenge and be used to provide sensory stimulation and train cognitive and psychomotor performance of crew without there being a requirement to undertake live operations. New technologies (e.g., artificial intelligence) may also be used to reduce communication delays and, thus, mitigate isolation consequences.

As frequently observed with space research, many new devices, technology, or stress management techniques, may, once tested in space, be efficiently applied to adaptation and performance on Earth in specific conditions. For example, during the sanitary crisis period, some results concerning adaptation to isolation and confinement obtained in space or in polar environments have been useful for people during confinement periods. Some operational or mental strategies identified and validated in space may be transferred to life on Earth in isolation, confined, and extreme conditions. In many instances, this might be in settings that have societal important e.g., climate scientists, defense and security personnel, and anti-poaching wildlife rangers. Since constraints on the design of such techniques can be largely relaxed for Earth applications compared to Space applications, more flexibility is a promise for wider applications for the public. Finally, the “space brand” exerts great charm on the public and can be a channel for the promotion of societal and psychological improvements. For example, pro-environment behaviors studied and reported by the astronauts may be mimicked on Earth; well-being promotion strategies that are currently developed for space explorations, such as certain mind-body techniques, can also be implemented on the planet, following the examples from the space context. There are therefore several environments in which behavioral space research can have a positive impact on Earth research and society, including educational, organizational, professional, and recreational contexts. To facilitate these benefits, the communication strategy implemented by all the involved actors (national agencies, private companies, astronauts…) should be mindful of these potential implications.

### Recommendations in short, middle, and long term

Table 1 reports the overarching categories representing the key open psychological research questions related to lunar and LDSE missions. Many of the open questions could be partly addressed in ground-based analogs. However, where the unique demands of missions beyond LEO and in deep space are relevant, ongoing research across various platforms will be needed. To effectively prepare for future LDSE missions, such as a Mars expedition, we suggest these questions should be addressed during a short to medium timeframe. There are certain unknowns that will only be elucidated over longer time periods and perhaps during a Mars mission itself. We recommend these timelines (3, 5, and 10 years) as a suggestion for addressing research gaps, although we are aware that research often requires longer times, so they do not necessarily correspond to expected research results.

**Table 1:** Suggested environment and timeline to address the fundamental research questions.

Open fundamental scientific questions (knowledge gaps)	Future space experiments and suitable environments (ground-based platforms, LEO (ISS), Moon, Mars, BLEO)	Timeline: short (3 years), medium (5 years), long (10 years)
**Basic issues of adaptation**		
What critical markers including the psychological and social variables and human behavior and performance competencies provide meaningful information on individual and team function during LDSE missions?	Ground-based platforms, LEO (ISS), Moon	Short, Medium
How do stressors unique to LDSE missions impact upon individual and team functions?	Ground-based platforms, LEO (ISS), Moon, Mars, BLEO	Short, Medium, Long
What impact do individual and team characteristics have upon adaptability and the function of the crew during space missions?	Ground-based platforms, LEO (ISS), Moon	Short, Medium
What resources are needed, and which approaches should be used to enable effective coping and self-regulation during and after return from LDSE missions?	Ground-based platforms, LEO (ISS), Moon	Short, Medium
How do spaceflight stressors, demographic criteria, individual and crew characteristics, and self-regulatory approaches, interact to impact upon individual and team function in space?	Ground-based platforms, LEO (ISS), Moon, Mars, BLEO	Short, Medium, Long
**Countermeasures**		
What modifications to selection and additional training is needed to ensure safe and effective individual and team function during missions beyond LEO?	Ground-based platforms, LEO (ISS)	Short, Medium
What resources and equipment need to be provided, and individual and team-based countermeasures developed, to enable effective monitoring, diagnosis, and support during LDSE missions?	Ground-based platforms, LEO (ISS)	Short, Medium
How should systems, architectures, and habitats be designed to ensure optimal individual and team function during missions beyond LEO?	Ground-based platforms, Moon, Mars, BLEO	Short, Medium, Long

### Countermeasure list

The countermeasures below have all been used to mitigate the psychological demands of spaceflight. However, beyond a few initial studies, there has been a relatively limited attempt to empirically test the impact of their application (see Table 2). Before these methods can be considered evidence-based further space and/or analog-based research would be needed.

**Table 2:** Available and to be developed countermeasures.

Countermeasure	Description	Pre	During	Post	Target (well-being and/or performance)
Mindfulness training^9^	Training and using skills to be present and attentive to physical and psychological states	X	X	X	W, P
Relaxation training^9^	Training and using skills to reduce arousal/activation levels	X	X	X	W, P
Adaptative team training^25,26^	Training and using skills to cooperate, coordinate and communicate effectively as a team	X	X		P
Mental skills and resilience training^27^	Training and using skills to withstand stress and maintain and optimize performance	X		X	W, P
Human behavior and performance competency training^28^	Training human behavior and performance competencies needed to be an effective crewmember	X			W, P
Simulation training^29^	Simulation training that mimics spaceflight technical tasks	X			P
Cognitive monitoring^30^	Regular assessment of cognitive function		X	X	P
Skill fade mitigation^29^	Simulation tests that maintain a baseline level of skill on technical tasks	X	X		P
Yoga, Ta Chi, exercise	Different exercises used as a stress reduction countermeasure	X	X		W
Journaling^31^	Keeping a diary—narrative therapy as a potential stress intervention		X		W
Virtual reality—sensory stimulation^29^	Using VR to access other environments	X	X		W
Virtual reality—social connection^32^	Using VR to increase relatedness in interpersonal interactions	X	X		W
Robotic assistance/conversational AI	Task and emotional support provided by smart robots/conversational AI	X	X		W, P
Private psychological conference	Speaking to an on-call psychologist		X		W, P
Private family conferences	Speaking to family and friends		X		W
Care packages	Receiving surprise care packages, treats, gifts		X		W
Performance debriefing	Team debriefs designed to capture lessons learned and improve function		X	X	P
Food and shared meals	Purposely making time for crew meals to build cohesion		X		W
Celebrations	Actively marking milestones and celebrating important dates		X		W
Crew discretionary events (non-public)	Crew time to engage in their own choice of events		X		W
Private homepages—crewmember interests	Somewhere crewmembers can upload and share their own experiences		X		W
Entertainment access	Providing access to required entertainment systems for spare time		X		W
Arts (music, photography)	Artistic opportunities as a way of using spare time		X		W

### Outlook and summary

This white paper reported the results of a consensus statement among experts invited by ESA about the existing knowledge gaps on behavioral and performance topics of space research. This is particularly timely, as exploration missions are moving from low orbit to deep space destinations, with new psychological and team challenges forthcoming. While this is a non-systematic review of these research gaps, the working group consisted of experts in space psychology, who have been engaged in space research for ESA. Pre-, during-, and post-mission challenges and research gaps were considered, referring to promising countermeasures, either with preliminary evidence of their effectiveness, or to be developed and tested. The results summarize a set of challenges and questions to be addressed, but also some potential answers that have already been provided by the scientific community over decades of space psychology research. New empirical evidence is required to address most of these gaps, collected with specifically designed studies. It must be noted that to address the contextual constraints (e.g., number of active astronauts, or available analogue environment), some of these gaps can be tackled with thorough reviews or white papers incorporating all extant research findings. While space psychology is likely to have an important future, researchers should also be mindful of previously developed knowledge.

## References

[1] Landon LB, Slack KJ, Barrett JD (2018). Teamwork and collaboration in long-duration space missions: Going to extremes. Am. Psychol.

[2] Kanas, N. Psychosocial Issues during an Expedition to Mars. In: *The New Martians. Science and Fiction*. (Springer, 2014). 10.1007/978-3-319-00975-9_2.

[3] Manzey D (2004). Human missions to Mars: new psychological challenges and research issues. Acta Astronaut.

[4] Suedfeld P (2005). Invulnerability, coping, salutogenesis, integration: four phases of space psychology. Aviat. Space Environ. Med..

[5] De la Torre GG (2014). Cognitive neuroscience in space. Life.

[6] ESA SciSpacE Expert Working Groups. *White Paper #13: Behavioural Health and Performance*. (European Space Agency, 2021).

[7] Oluwafemi FA, Abdelbaki R, Lai JC-Y, Mora-Almanza JG, Afolayan EM (2021). A review of astronaut mental health in manned missions: potential interventions for cognitive and mental health challenges. Life Sci. Space Res..

[8] Sipes, W., Holland, A., Beven, G. Managing Behavioral Health in Space. In: *Handbook of Bioastronautics*. (eds Young, L. R. & Sutton, J. P.) (Springer, 2021). 10.1007/978-3-319-12191-8_118.

[9] Pagnini F, Phillips D, Bercovitz K, Langer E (2019). Mindfulness and relaxation training for long duration spaceflight: Evidences from analog environments and military settings. Acta Astronaut.

[10] Farand A (2001). The code of conduct for international space station crews. ESA Bull..

[11] Gabriel G (2012). Future perspectives on space psychology: recommendations on psychosocial and neurobehavioural aspects of human spaceflight. Acta Astronaut.

[12] Clement, G. R. et al. Spaceflight Standard Measures. In *Human Research Program Investigators' Workshop (HRPIPW)* (No. JSC-E-DAA-TN77371) (2020).

[13] Wenzel, E. M. Standard Measures for Use in Analog Studies, ISS, and Research for Long-Duration Exploration Missions (No. NASA/TM-20210021987) (2021).

[14] Dinges, D. S*tandardized Behavioral Measures for Detecting Behavioral Health Risks during Exploration Missions*. https://www.nasa.gov/mission_pages/station/research/experiments/explorer/Investigation.html?#id=7537 (2017).

[15] Kanas, N. & Manzey, D. *Space psychology and psychiatry*, vol. 16 (Springer, 2008).

[16] Kanas N (2011). High versus low crewmember autonomy during a 105-day Mars simulation mission. Acta Astronaut..

[17] Fischer, U. & Mosier, K. in *Proc. Human Factors and Ergonomics Society Annual Meeting,* 164–168 (SAGE Publications Sage CA) (2020).

[18] Goemaere S, Van Caelenberg T, Beyers W, Binsted K, Vansteenkiste M (2019). Life on mars from a self-determination theory perspective: how astronauts’ needs for autonomy, competence and relatedness go hand in hand with crew health and mission success-results from HI-SEAS IV. Acta Astronaut..

[19] Sandal GM, van deVijver FJ, Smith N (2018). Psychological hibernation in Antarctica. Front. Psychol..

[20] Palinkas L (1986). Health and performance of Antarctic winter-over personnel: a follow-up study. Aviat., Space, Environ. Med..

[21] Suedfeld P (2010). Historical space psychology: early terrestrial explorations as Mars analogues. Planet. Space Sci..

[22] Clément GR (2020). Challenges to the central nervous system during human spaceflight missions to Mars. J. Neurophysiol..

[23] Slack, K. J. et al. Risk of Adverse cognitive or behavioral conditions and psychiatric disorders: Evidence report (No. JSC-CN-35772) (2016).

[24] Landon, L. B. Risk of Performance and Behavioral Health Decrements Due to Inadequate Cooperation, Coordination, and Psychosocial Adaptions within a Team. https://ntrs.nasa.gov/api/citations/20220007465/downloads/Evidence%20Report%20-%20Team%202022%20FINAL4PUB%20rev%201.docx.pdf (2022).

[25] Croitoru, N., Bisbey, T. M. & Salas, E. in *Psychology and Human Performance in Space Programs*, 81–99 (CRC Press, 2020).

[26] Landon, L. B. & O’Keefe, W. S. in *Building Intelligent Tutoring Systems for Teams*, vol. 19, 279–298 (Emerald Publishing Limited, 2018).

[27] Shelhamer M (2016). A call for research to assess and promote functional resilience in astronaut crews. J. Appl. Physiol..

[28] Buckle, S., Peldszus, R. & Bessone, L. in *Space Safety is No Accident*, 303–312 (Springer, 2015).

[29] Garcia, A. D., Schlueter, J. & Paddock, E. Training Astronauts using Hardware-in-the-Loop Simulations and Virtual Reality. AIAA 2020-0167. AIAA Scitech 2020 Forum. 10.2514/6.2020-0167 (2020).

[30] Korovin I, Klimenko A, Kalyaev I, Safronenkova I (2021). An experience of the cognitive map-based classifier usage in astronaut’s emotional state monitoring. Acta Astronaut..

[31] Stuster, J. *Behavioral issues associated with long-duration space expeditions: review and analysis of astronaut journals: experiment 01-E104 (Journals)*. (National Aeronautics and Space Administration, Johnson Space Center Houston, 2010).

[32] Salamon N, Grimm JM, Horack JM, Newton EK (2018). Application of virtual reality for crew mental health in extended-duration space missions. Acta Astronaut..

